# P09-03 Costing the economic burden of sedentary behaviors in France

**DOI:** 10.1093/eurpub/ckac095.133

**Published:** 2022-08-29

**Authors:** Antoine Noël Racine, Irène Margaritis, Martine Duclos, François Carré, Anne Vuillemin, Gautier Christèle

**Affiliations:** 1 Pôle Ressources National Sport Santé Bien-Etre, French Ministry of Sports and the Olympic and Paralympic Games, Vichy, France; 2 French Agency for Food, Environmental and Occupational Health & Safety (ANSES), Maisons-Alfort, France; 3 Service de Médecine du Sport et des Explorations Fonctionnelles, CHU Clermont-Ferrand, Clermont-Ferrand, France; 4 INRAE, UNH, F-63000, Université Clermont Auvergne, Clermont-Ferrand, France; 5 LTSI INSERM, U1099, University of Rennes 1, Rennes, France; 6 Department of Sport Medicine, Pontchaillou Hospital, Rennes, France; 7 Université Côte d'Azur, LAMHESS, France; 8 Sport Policy Development Office, French Ministry of Sports and the Olympic and Paralympic Games, Paris, France

**Keywords:** sitting time, cost analysis, health expenditures, burden of disease

## Abstract

**Background:**

There is strong evidence showing that sedentary behaviour (SB) increase the risk to develop several chronic diseases and to premature death (Chau et al., 2015). A dose response relation is observed with a more marked risk when people spend more than 7 hours/day in sitting position (Ekelund et al., 2019). The study INCA 3 on the lifestyle habits of French population indicated that 40% of people between 18 and 79 years had a high risk for health conditions with more than 7 hours of daily SB (ANSES, 2017). The economic consequences of this risk have never been evaluated. The aim of this study was to estimate the economic burden of SB-related diseases in France.

**Methods:**

From meta-analysis or large cohorts based on individual SB time, we identified relative risk (RR) to develop cardiovascular disease (CVD), colon cancer, breast cancer and all-causes premature death after co-variables adjustments including physical activity. From RR and prevalence of SB time in France, a population attributable fraction approach was used to estimate the yearly number of cases for each disease. Data from the national health insurance were used to calculate the annual average costs per case for each disease. Then, disease-specific and total health-care costs attributable to prolonged SB time were calculated. Indirect costs for private sector and households were calculated in a second stage.

**Results:**

In France, 66 528 premature deaths/year appear related to a daily SB time ≥ 8,6 hours Each year prolonged SB cost 559 millions € for the national health insurance, including 359 millions € for CVD (≥ 10 hours of daily SB), 170 millions € for breast cancer (≥ 6 hours of daily SB), and 31 millions € for colon cancer (≥ 5 hours of TV/day).

**Conclusions:**

These preliminary results showed that many deaths could be avoided by reducing prolonged SB prevalence in France. Moreover, direct health-care costs attributable to SB related diseases represent a high economic burden for the French health system. To address this issue, strong responses should be implemented to tackle SB, complementary to physical activity promotion.

